# Advanced nanocomposites for microplastic remediation: a critical review of materials, mechanisms, and scalability challenges

**DOI:** 10.1039/d6ra02233b

**Published:** 2026-05-18

**Authors:** Veena C. G., Chitra D.

**Affiliations:** a School of Chemical Engineering, Vellore Institute of Technology Vellore Tamil Nadu 632014 India dchitra@vit.ac.in

## Abstract

Microplastic pollution has become a widespread environmental problem, driving the need for remediation strategies that are not only effective but also sustainable. For this, advanced nanocomposites offer a transformative substitute through their customizable functionality and high surface reactivity. This review offers a critical and narrative assessment of recent advances in nanocomposite-based microplastic remediation, with a focus on materials like magnetic, carbon-based, polymeric, and photocatalytic, *etc.* Although reported removal efficiencies vary widely, ranging from roughly ∼73% to as high as ∼99.96%. Specifically, these statistical values are derived from the fundamentally different metrics, such as mass loss, surface degradation, particle reduction, and total organic carbon (TOC) removal. Because these are not directly comparable, they may complicate any honest evaluation of true performance and lead to an overestimation of the material efficiency. By systematically examining structure and property relationships, this review highlights how the material parameters, such as surface area, density of functional groups, band gap, and heterojunction formation, govern adsorption capacity and dictate degradation pathways. It also identifies major limitations in current research, including the absence of standardized testing protocols, insufficient attention to complete mineralization of microplastics, and a general lack of assessment regarding real-world scalability. Looking ahead, the review argues for unified evaluation frameworks, clearer mechanistic understanding, and testing conditions that reflect actual environmental scenarios; only then can nanocomposite technologies be meaningfully compared and eventually deployed in practice for microplastic remediation.

## Abstract 2

Microplastics cause serious environmental and health issues. Conventional water treatments exhibit limited removal efficiency, whereas nanocomposites enhance removal *via* different methods, yet sustainable optimization remains necessary.

## Graphical abstract

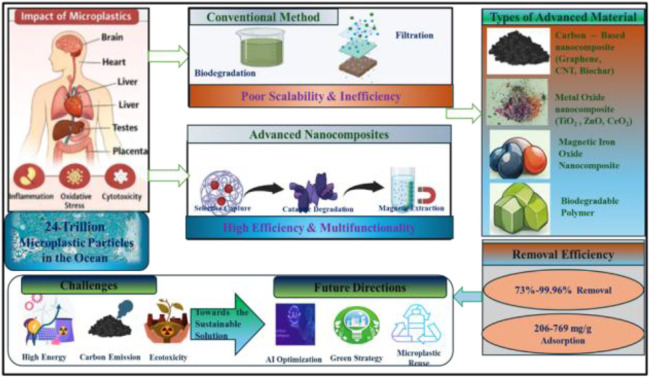

## 1. Introduction

**Fig. 1:**
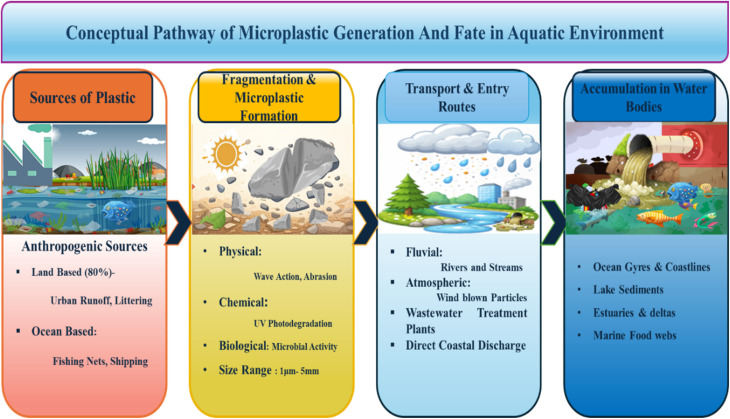
Schematic illustration of the microplastic pathway in the aquatic environment.

In the 20th century, plastic materials represented one of the more consequential legacies of innovation because of their durability, versatility, and low cost.^1^ According to the global production statistics, in the year 1950, annual output had escalated from approximately 1.5 million metric tonnes to current estimates approaching 500 million metric tonnes.^2,3^ This exponential growth of plastic material has been accompanied by a very dangerous accumulation of both micro/nano-plastic waste in land and aquatic ecosystems.^4^ The microplastics are usually defined as plastic particles smaller than 5 mm in size.^5^ These are generally recognized as pervasive pollutants in different types of aquatic environments, such as in freshwater, marine, and even remote polar waters.^6,7^ This is due to their ability to be transported *via* interrelated environmental pathways like soil, water, and air.^8,9^ Mainly, these microplastics originate from two sources: first, from the use in products like microbeads and synthetic fibres,^10^ and second, from the breakdown of larger plastic fragments through any other mechanisms like solar radiation, UV radiation, mechanical weathering, and also by biological activity (Fig. 1).^11–13^ From these sources, microplastics are dispersed through the water column, surface, and sediments.

Microplastic contaminants in global environments have been detected with increasing prevalence, in a range of aquatic matrices from surface waters of the North-East Atlantic to deeper international freshwater systems.^14^ The mean concentration of 4.9 particles per liter, identified through quantitative testing of influent water at treatment facilities in England and Wales, demonstrates the ubiquity of these pollutants in primary sources of water.^15,16^ Although modern wastewater infrastructure can reach removal efficiencies of nearly 99.9%, such benchmarks are insufficient to guarantee the absolute exclusion of the smallest particulate fractions.^16,17^ Generally, the microplastics themselves are very diverse in the world, and they exist in the form of fibers, films, fragments, and spheres.^18^ These are all made from polymers like polyethylene (PE), polypropylene (PP), polyethylene terephthalate (PET), and polystyrene (PS). According to their structural morphology and chemical reaction, determine the interaction mechanism within aquatic systems.^19,20^

Microplastic materials play a crucial role in environmental pollution. Due to their resemblance to natural food products, ingestion by diverse aquatic organisms at multiple trophic levels.^21,22^ This can lead to a very dangerous threat to the environment as well as the organisms in this world. If it continuously enters the body of a human being or any living thing, it can cause both physical and mental issues. The toxicological implications arising from microplastic contamination extend considerably beyond simple mechanical interference with digestive processes.^23^ When examining tissues from aquatic animals exposed to microplastics over extended periods, they consistently observe inflammation in the gut lining. This inflammation shows up as an accumulation of immune cells, shortening of the finger-like projections that absorb nutrients, and breakdown of the protein connections that maintain the gut barrier. For humans, this will cause physical problems like blockages, tenderness, impaired feeding, and decreased energy reserves. The exposure of microplastics induced cytotoxicity by triggering excessive ROS production. This will lead to lipid peroxidation and subsequent structural and functional damage of cellular membranes and organelles.^23^

In the aquatic environment, the microorganisms, like algae, act as the carriers of these microplastic materials. These microplastics are not just physical pollutants; they can also cause chemical contamination, as well as serve as a transport path for toxic substances.^24,25^ In short, in the aquatic environment, microplastics signify both as a physical and chemical hazard. These give importance to addressing their impacts and sources worldwide.^23^ Moreover, microplastics serve as vectors for toxic substances, adsorbing hydrophobic pollutants such as PCBs, PAHs, and heavy metals from surrounding water and facilitating their bioaccumulation and trophic transfer.^26^ This leads to the importance and urgency of microplastic research, which has unquestionably driven rapid operational advancements.

Lack of proper understanding of the degradation process was the main limitation at the initial stage of the microplastic research.^27^ These limitations pose additional challenges for microplastic research, especially for nanoplastics.^28,29^ This nano/microplastic went largely unnoticed.^30^ To overcome this limitation, researchers introduced a wide range of advanced analytical techniques, such as UV-Spectroscopy, Raman spectroscopy, and Fourier Transform Infrared (FTIR) spectroscopy, *etc.*^31^ These analytical methods are later being predominantly effective for particles that are smaller than 0.1 mm in complex water sources like tap water.^32^ Because there are no standard testing methods available for the comparative studies for this.^33^ Even if each technique has its own advantages, it also carries inherent constraints. In this FT-IR analysis becomes unreliable for particles below 20 µm due to diminished signal-to-noise ratios and scattering artifacts.^34^

However, in the case of dark or weathered particles containing carbon black, the detector response renders spectral interpretation vague.^35^ Considering the sample preparation of readily processed particles, more care should be taken about their drying, flattening, and organic matter removal. Because it gets easily biased by the material.^36^ In the case of Raman spectroscopy, while offering superior spatial resolution, it suffers from fluorescence interference originating from biological residues and polymer additives, often obscuring characteristic spectral signatures.^37^ While the thermal degradation of labile polymers under prolonged laser exposure may alter spectral features or destroy small particles before identification. Alternative thermal methods like pyrolysis-GC/MS enable polymer confirmation, but they destroy the morphological information and preclude particle record.^38^ Even if it has limitations, after the discovery of these techniques, progress has been visible to a notable extent. After this invention, most modern water treatment plants still struggle to remove these minute contaminants completely from the drinking water.^31^

**Fig. 2:**
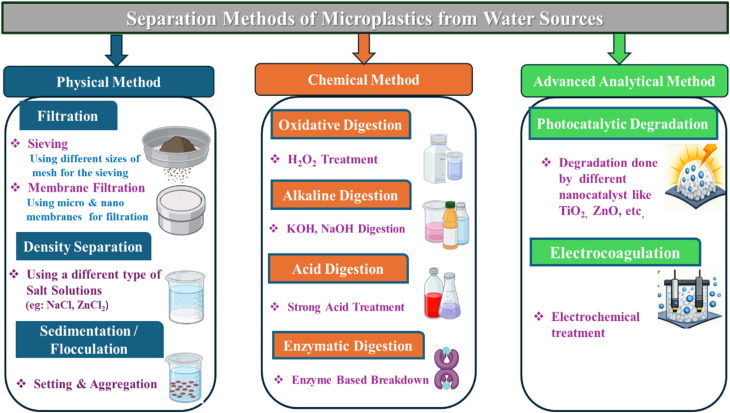
Different separation methods of microplastics from water sources.

For the identification, conventional treatment approaches operate in three stages: primary screening and sedimentation (∼75% removal of particles >300 µm), secondary biological treatment (∼90% removal through biomass entrapment), and tertiary advanced filtration including ultrafiltration, nanofiltration, and reverse osmosis (>99% removal).^38–44^ Complementary physicochemical methods, such as coagulation–flocculation–sedimentation and dissolved air flotation, further enhance particle aggregation and removal (Fig. 2). For detailed analysis of this sample, mass spectrometry and pyrolysis-gas chromatography/mass spectrometry (Py-GC) are used. These characterization techniques provide molecular-level information on the thermal decomposition of the microplastics.^38^ The current and most useful method is the combination of these physical (microscopy) and chemical (spectroscopy) techniques. This is used to ensure the accurate identification and quantification of microplastics in environmental samples.^24^

The challenges caused by microplastics in aquatic environments, sustained research and technological development remain essential. Human activities cause the extensive share of microplastic contamination. Even though wastewater facilities are engineered to remove various pollutants, their capacity to retain the smallest microplastic particles remains limited. As a result, these treatment gaps enable the continual release and buildup of microplastics in natural water systems.^45–49^ The widespread use of microplastic material in the ecology makes them hard to measure or count. These limitations have driven interest in next-generation materials capable of addressing microplastic pollution through fundamentally different mechanisms.^50^ The appearance of nanocomposite materials with large surface area, controllable chemical and physical functionalities, and enhanced adsorption compared to pure carbon-based materials has provided an attractive alternative.^51^ These advanced materials can be designed to specifically interact with surfaces of microplastics, where efficient physical adsorption and even, in some cases, chemical degradation is facilitated.^42^

Nanomaterials have been receiving significant interest in environmental science owing to their high surface area and tailorable surface functionalities. These features endow them with unique physicochemical properties, enhancing their utility in catalysis, remediation, and sensing applications.^52^ The main reason for this is the elevated specific surface area of nanoscale constituents, which typically range from 100 to over 1000 m^2^ g^−1^ for materials such as GO and carbon nanotubes.^53^ This provides substantially greater interfacial contact area per unit mass compared to granular activated carbon or polymeric membranes.^54^ Second, the high density of surface-active sites, including oxygen-containing functional groups on biochar and graphene derivatives, facilitates multiple interaction modes with microplastic surfaces like electrostatic attraction, hydrophobic segmentation, π–π stacking with aromatic polymer domains, and hydrogen bonding with polar functionalities.^55^ Third, nanoscale dimensions enable access to microplastic surface features and gaps inaccessible to larger conventional particulates, enhancing capture efficiency for irregularly shaped fragments.^56^ Fourth, multifunctional architectures incorporating photocatalytic or magnetic components enable simultaneous adsorption and degradation or facile post-treatment recovery.^55–57^ These capabilities are absent in conventional single-function material. However, these performance advantages observed under controlled laboratory conditions often diminish in complex environmental matrices where natural organic matter competes for active sites and ionic strength modulates electrostatic interactions.^58^

Notable material classes under investigation include carbon-based nanomaterials (graphene oxide, carbon nanotubes, biochar), metal-oxide photocatalysts (TiO_2_, ZnO, CeO_2_), magnetic iron oxide systems that enable facile recovery, and biodegradable polymer hybrids that offer sustainable end-of-life profiles.^59–61^ These advanced nanocatalysts are not limited to aqueous environments. In air purification, they help break down volatile organic compounds and some greenhouse gases.^62^ In soil systems, nanoscale zero-valent iron was found to be an effective reagent for the remediation of chlorinated solvents and pesticide residues.^63^ With the development of nanomaterials in environmental technology, there has been a standard shift towards higher efficiency, improved selectivity, and resource recovery, making them a vital material for next-generation environmental devices.^64^

Several comprehensive reviews have recently addressed microplastic remediation through nanomaterial-based approaches, yet this work occupies a distinct analytical role. Some reviews focus on biochar derived from agricultural waste and its composites, discussing how feedstock type, production conditions, and surface modifications affect the adsorption.^65,66^ Some other studies examine the nanomaterial-enhanced biochar systems and explain the mechanisms, such as π–π interactions and magnetic recovery, while also offering practical application strategies.^65,67^ Another study explains that the photocatalytic approaches, particularly those using natural precursors or porous crystalline materials, have also been thoroughly reviewed in major environmental journals.^66^ Although these works often include waste restraint, technological & economic factors, and life-cycle thinking.^66,68^ A few studies explore coordination chemistry and nanocomposite methods for water treatment, providing detailed mechanistic insights.^69^ Still, they rarely focus on the ecological impacts or long-term fate of used nanomaterials.^67^ Overall, previous reviews have largely been material- or mechanism-specific, and few have attempted a broad, comparative analysis across different nanocomposite types within a single framework.^65^ This review critically examines four major categories of nanocomposite materials for microplastic remediation, evaluating their synthesis routes, underlying mechanisms of action, and demonstrated removal efficiencies. Particular attention is keen on performance metrics under environmentally relevant conditions, scalability challenges, and sustainability considerations, including life-cycle impacts and secondary pollution risks. It also synthesizes current advancements to establish a framework for developing ecologically sustainable remediation technologies, while identifying critical research gaps to guide future studies.

## 2. Route to nanocomposite fabrication

**Fig. 3:**
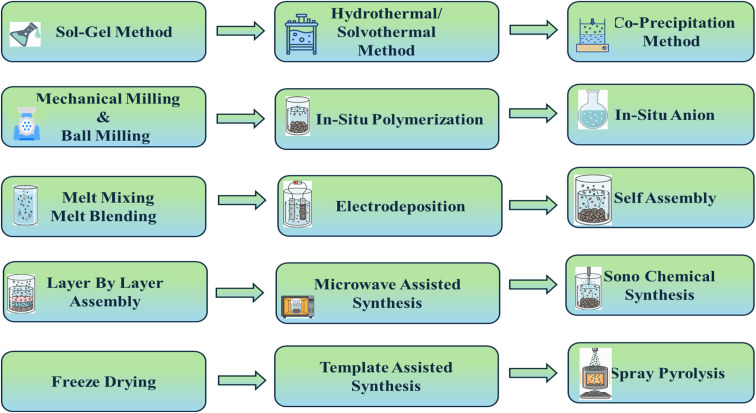
Diverse methodologies for the structural assembly of nanocomposite materials.

The synthesis of nanofilms, nanomaterials, and nanocomposites is broadly classified into 4 methods: chemical, physical, biological, and advanced (hybrid) methodologies (Fig. 3). In these methods, the selection of a suitable method depends on the proposed application of the material. Each method has its own advantages and limitations. The chosen synthesis method will be used to characterize the final product's nature, structure, properties, and scalability.^70^ Physical methods like melt blending, high-energy ball milling, and extrusion rely on mechanical force and thermal energy to achieve component incorporation.^71^ This approach is often mentioned as a top-down strategy and is also prized for its operational simplicity, high output, and direct scalability for bulk-scale manufacturing.^72^ However, one of the significant drawbacks is that these methods cannot produce nanoscale materials. Moreover, they often fail to resolve phases and ensure homogeneous dispersion. This may lead to issues like agglomeration and poor interfacial adhesion.

The validity of a selected synthesis route is evaluated based on the resulting material's morphology and feasibility. Three key factors guide this evaluation. First, dispersion homogeneity, the uniform distribution of nanomaterial within the matrix, is essential, as agglomerates tend to function as defects rather than reinforcements.^73^ The second one is the physical morphology of the material; preserving their aspect ratio and structure is essential for effective load transfer or network formation.^74^ Last one is the interfacial interactions between the absorbent surfaces and the matrix, which govern how effectively stress, electrons, or heat are transferred across the boundary.^75^ Consequently, the degree of control achieved over these parameters, such as dispersion, morphology, and interfaces during synthesis, ultimately orders the performance ceiling of the final nanocomposite. Hence, influencing its sustainability and reliability in demanding real-world applications, such as biomedical devices, aerospace components, and flexible electronics.^76^ Although the broader materials literature documents numerous synthetic methodologies, the present discussion restricts its scope to those fabrication approaches for which empirical evidence demonstrates tangible consequences for microplastic sequestration efficiency, catalytic transformation activity, or post-treatment material recovery.

**Fig. 4:**
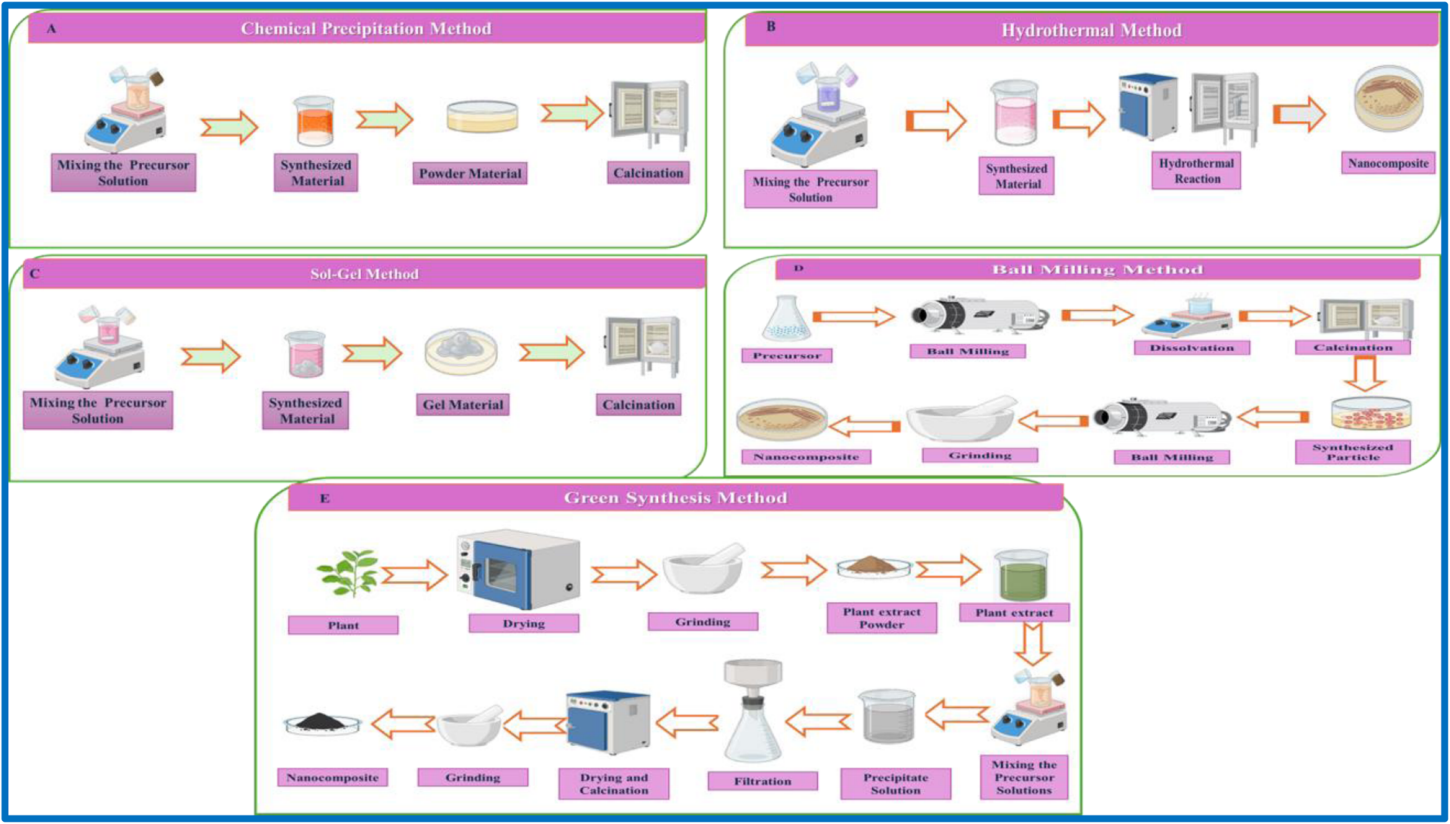
Schematic illustration of five common synthesis routes for nanocomposites used in microplastic remediation: (A) chemical precipitation, (B) hydrothermal synthesis, (C) sol–gel, (D) ball milling and (E) green synthesis.

The chemical synthesis method is typically categorized as a bottom-up technique because these materials are built from the molecular precursors.^77^ The type of synthesis contains techniques like co-precipitation, *in situ* polymerization, sol–gel process, and chemical vapor deposition. All these methods allow the subtle control over the particle size, morphology, and distribution on the synthesised material.^78^ This will create a strong chemical bond between the environment and the dispersed phase, which is the main advantage of these methods. These will significantly enhance the stress transfer and composite performance.^79^ However, this control requires lots of criteria, such as specialized precursors and high temperatures, *etc.* This will lead to high costs and a complex synthesis method.^80^ To overcome this issue, an alternative method is green synthesis. This offers a sustainable method by using plants or microorganisms as eco-friendly capping and reducing agents. The main principles of Green Chemistry are that the minimum quantity of hazardous waste is produced with the use of gentle solvents and precursors, and it is also renowned for its energy efficiency.^81^Fig. 4 shows the schematic illustration of five common synthesis routes for nanocomposites used in microplastic remediation. Remarkably, there is no single method that is universally greater. Consequently, nowadays lots of hybrid approaches have been developed to synergize the strengths of multiple techniques. For example, a physical pre-dispersion step may be combined with *in situ* polymerization to optimize both dispersion quality and processing efficiency.^82^

### 2.1 Nanocomposite synthesis: relevance to microplastic remediation

#### 2.1.1 Fabrication of polymer-matrix nanocomposites

In the field of materials science, the situ polymerization technique is widely recognized as a transformative method for the synthesis of nanocomposites. The fundamental principle of this method is the uniform dispersion of the molecular precursor within the monomer medium before polymerization. This strategy directly addresses two critical encounters in nanocomposite fabrication. The first one is to achieve a homogeneous nanoparticle dispersion, and the second one is the establishment of a strong interfacial bonding between the composite and the resulting polymer matrix.^83^ Which reduces the agglomeration of material as compared to other methods. Subsequently, the nanocomposite exhibits significant enrichments in its key properties, such as an increase in ionic conductivity and superior mechanical toughness, *etc.* These improvements are credited to the continuous ion-conducting pathways and strong filler-matrix interactions facilitated by the uniform microstructure.^81^

To address this, researchers optimize parameters like mixing time, temperature, and shear rate.^84^ Such characteristic performance is critical for advanced applications, particularly for energy storage devices and flexible electronic systems.^85^ One of the best examples of this method is the formation of gold nanoparticles induced by γ-radiation. This radiation-based method is superior to other methods because it's a very eco-friendly way to make nanoparticles and composites. Instead of using different toxic chemicals, radiation is used to control nanoparticle formation. Mainly, these materials are environmentally safe and highly effective for biomedical sensing and advanced catalysis. Moreover, this method is easy for industrial production.^86^ One of the major trends in creating these advanced materials is the combination of strong chemical bonds with *in situ* polymerization. One of the best examples of this is the grafting of polyaniline into a graphene sheet directly by using a coupling agent like perfluorophenyl azide. This will result in a graphene-based nanocomposite with properties of high durability and conductivity. The chemical bond is the main reason for this performance advancement. This will create a direct and strong connection between the graphene and the polymer. This also acts as a bridge between them and prevents the separation from taking place due to degradation.^87^ This powerful combination allows researchers to create modified, high-performance materials for advanced applications like electronics, sensors, and storage devices. In addition, surface modification of nanoparticles is often required to enhance compatibility with the polymer. The uniform dispersion of the nanomaterial on the polymer solution makes this method a more practical route to commercial production.^84^

#### 2.1.2 Preparative routes for metal-oxide photocatalysts

The sol–gel method is widely used for the preparation of metal oxide nanocomposites. Mainly because of its low operational temperature and integrates readily with organic components.^88^ This process involves mixing a metal alkoxide precursor solution into a “sol”, which means a colloidal suspension. This undergoes further hydrolysis and condensation to form a 3D “gel”.^88^ One of the major advantages of this technique pure, uniform material, and it has high control over its surface area, porosity, and homogeneity. This will make them highly performant for applications like catalysis, coatings, *etc.*^89^ The next method is the co-precipitation or chemical precipitation. This is the method widely used in the research field because it is a very cost-effective approach in the bottom-up method.^90^ In this process, the chemical reaction takes place between the metallic precursor and the precipitating agent very rapidly and forms the nanoparticles or composites.^90,91^

In the entire chemical process, the hydrothermal method is the foremost one. This is the broadly used technique for synthesising high-quality nanoparticles. In this method, the total reaction takes place in the hot, pressurized, sealed container called an autoclave.^92^ The high temperature and pressure enhance the solubility and reactivity of the precursor. This will allow the direct synthesis of crystalline materials without needing further processes, such as high-temperature calcination.^92^ This versatile method is a supporting factor for modifying the size, shape, and phase of advanced materials like nanowires, nanocomposites, and nanorods, *etc.*^92^ Researchers commonly synthesize carbon-nanotubes with zinc oxide as a nanocomposite by using the sol–gel method.^93,94^ In this, the zinc oxide layer will deposit flawlessly on the surface of the CNTs in a uniform manner.^94^ This flawless uniform coating gives a better charge transfer between the two materials.^94^ This makes them highly effective and is also used as a photocatalyst for water purification.^95^ It also allows adjusting the properties like particle size, porosity, phases, *etc.*^93^ These morphologies are very important for the nanocomposite materials because it is very essential for applications like storage battery, photocatalysis.^93^

From a mechanical and practical perspective, there are various fabrication techniques available for the polymer-based nanocomposite. In solution casting remains the preferred method for the synthesis of polymer-based nanocomposite films because of its simplicity and high versatility.^96^ In this method, a polymer precursor solution is dissolved in a suitable solvent, which is mixed with a nanomaterial to create a dispersion of the solution. After the evaporation of the solvent, there will be a solid nanocomposite film will remain.^96^ Researchers are particularly using this method in the field of sensors and membranes. Because it allows for the specific control over the thickness of the final thin film. Moreover, this method easily allows for changing its properties and making new materials on a small scale because it's highly adaptable and perfect for fundamental studies.^97^ Anyhow, the solution casting is a straightforward and effective method for the synthesis of polymer-based films in the laboratory. But in the case of industrial base production is very difficult.^97^ This is due to small-scale production; the solvent evaporation and drying are simpler, but in the bulk production, controlling the parameters like temperature, thickness, air flow, leftover solvent, contaminations, defects, *etc.*, is very challenging.^97^

#### 2.1.3 Fabrication of ceramic or metallic nanocomposites

One of the popular top-down techniques is mechanical milling, which is mainly used for the production of ceramic or metallic nanocomposites.^98^ This method is highly scalable and cost-effective, and eco-friendly, when compared to other methods. In this process, high-energy ball mills are used for the grinding of the bulk material into a smaller size, and also allow the large-scale production.^98^ This also has some limitations; the main limitation is the contamination because the ball mill normally uses iron or tungsten balls, which may react with the desired material.^98^ The next one is that the grinding jar may also cause contamination, which will lead to the formation of low-purity products. In this process, no one can predict the precise particle size and morphology of the produced material, because it will be a heterogeneous powder mixture having diverse shapes and sizes.^98^ Even though it has so many limitations, this method is still used in industrial-scale manufacturing, but it is less suitable for high-performance applications.^96,98^

Another advanced method for the synthesis of these ceramic or metallic nanocomposites is the vapour-phase deposition technique. There are 2 types of deposition techniques available, 1st is chemical vapor deposition, and the 2nd is physical vapor deposition.^99^ In these methods, which allow the atomic level control over the composition, uniformity, and thickness of the thin film. It has extraordinary precision over the material properties, which is the main reason for the use of high-performance applications like sensors and solar cells.^100^ Still, these methods have their own difficulties, such as the fact that they need specialized equipment, huge capital investment, a complex operational process, and a very slow reaction time.^101^ Besides, this method is fundamentally designed for surface coatings rather than producing structural, bulk materials. This limits its application in environmentally related mass production.^98^

#### 2.1.4 Fabrication of advanced functional layered nanocomposite

One of the additional methods is the electrochemical deposition and layer-by-layer assembly technique. This is widely used for the synthesis of advanced functional layered nanocomposites.^102^ In this method, the thin film was prepared by using an electric current on the metal ions and nanoparticles. The characteristics of these materials can be altered by adjusting parameters like concentration and voltage, *etc.*^103^ Similarly, the method of layer-by-layer assembly offers flexible nanoscale design capabilities by relying on the successive adsorption of oppositely charged components.^103^ These two techniques have their own advantages in producing innovative films because both methods enhance the specific sensing ability and conductivity. This makes these synthesized films ideal for energy-based applications like batteries, sensors, or diodes.^102^ Moreover, the intensive mixing techniques that are used in the laboratory for consistently spreading throughout the mixture do not apply to large-scale production. For bulk production, the nanoparticles tend to clump together due to agglomeration. This heterogeneous structure makes the nanofilms unreliable and unsuitable for commercial use.^104^

#### 2.1.5 Biologically mediated synthesis

Currently, materials science is undergoing significant changes in biomedical applications.^105^ That is moving away from the harsh and traditional chemical synthesis towards an eco-friendly, biological method.^106^ Due to environmental concerns and the need for an eco-friendly approach, this method incorporates materials like plant extract and microbes, *etc.* This will produce a highly efficient, sustainable, and reusable catalyst.^106^ The main significant advantage of this eco-friendly green synthesis is that the precursor materials are readily available and naturally coat. This makes them suitable for desired applications like biosensing, drug delivery, *etc.* One of the best examples of this is the synthesis of reduced graphene oxide/cuprous oxide (rGO/Cu_2_O) nanocomposite using lactulose.^106^ This wonderful evolution is an excellent combination of environmentally friendly methods with high-performance material.

Current research increasingly focuses on synthesizing hydrogels from naturally occurring biodegradable materials, such as chitosan (biopolymer) known for its high-water content and suitability for medical applications.^107^ Ongoing efforts aim to develop hybrid materials by integrating nanoparticles, including carbon-based nanomaterials, into these natural hydrogel matrices. Such hybrids exhibit enhanced structural integrity and mechanical strength while preserving the non-toxic and biodegradable properties.^108^ In the case of regenerative medicine, this combination is very critical for advancing performance and sustainability. This trend is widely used in green synthesis and boosts an interdisciplinary approach. The main goal is to develop advanced, sustainable materials that are safe and effective for real-time use in the clinical field. This marks a critical advancement in both environmental and biomedical technology.^108^

### 2.2 Interrelationship of synthetic protocol and remediation efficacy

Microplastic removal from the aquatic environment using nanocomposites is primarily governed by two mechanisms, physicochemical adsorption and physical entanglement.^109^ Initially, common plastics such as PE and PP are hydrophobic; they are naturally attracted to the hydrophobic surfaces of nanomaterials, such as GO or carbon nanotubes.^110^ Specifically, molecular interactions, such as π–π stacking and van der Waals forces, are enhanced and occur between the aromatic rings of the microplastics and the surface of the nanomaterial.^110^ Although van der Waals forces are very weak, they become strong stabilizers due to the high surface area of nanomaterials.^111^ For microplastic removal, physical entrapment acts as a crucial, nonselective mechanism. The nanocomposite interacts with porous materials such as membranes, zeolites, and metal–organic frameworks (MOFs), forming a complex network that acts as a molecular sieve. This structure enhances the mechanical retention of microplastics based on size, effectively filtering out particles larger than the pore openings.^109^ The efficacy of both adsorption and entrapment is dynamically influenced by environmental parameters, including pH, which alters surface charges, and ionic strength, which can screen electrostatic interactions.^109^

One of the best examples of this approach is the synthesis of silicon dioxide (SiO_2_) nanoparticles with polyethylene oxide (PEO) composite *via* a non-hydrolytic sol–gel route. In this process, the reaction takes place in the presence of the PEO, so the interaction of the nanomaterial and the polymer will be at the molecular level. This process is based on Lewis's acid-catalysed condensation reaction of tetraethyl orthosilicate (TEOS) within the controlled environment of PEO chains. This will result in the controlled nucleation and growth of silica nanoparticles.^112–116^ To improve this unreceptive capture method, the nanocomposites are now designed for active redress and efficient retrieval. One of the prominent methods for this is flocculation, where the nanocomposites are reacted with the biopolymers, such as chitosan.^107^ These biopolymers use the surface functional groups like –OH, –NH_3_ to form the bridge and create the large, settleable flocs that can easily be removed from the water.^117^ In this reaction, materials capture microplastics *via* an adsorption or flocculation method.^118^ The captured microplastics can be easily removed or retrieved with the help of an external magnet, followed by the traditional filtration method.^119^ Beyond these traditional extraction methods, photocatalytic degradation offers a destructive pathway for the removal of microplastics from the wastewater.^120^ When the light irradiation takes place, semiconductor nanocomposites like TiO_2_ or g-C_3_N_4_ generate reactive oxygen species (ROS), including hydroxyl radicals (˙OH).^121^ These ROS mineralize the microplastics into gentle end products like CO_2_ and H_2_O.^122^ This approach is very effective for addressing this environmental subject.

## 3. Major nanocomposite categories for microplastic remediation

Nowadays, the advanced nanocomposite materials are increasingly relied upon for microplastic remediation. Because they outstrip traditional methods in capturing, separating, and degrading pollutants.^123^ For instance, the magnetic photocatalytic nanocomposites can simultaneously adsorb microplastics and degrade them when the light exposure takes place.^124^ Similarly, flocculant-functionalized magnetic hydrogels aggregate microplastics from water for magnetic separation and following treatments.^125^ These materials achieved high-capacity adsorption, rapid magnetic recovery, *in situ* photocatalytic mineralization, and superior removal efficiency while minimizing secondary pollutants.^126,127^ In the case of photocatalytic nanocomposites, such as titanium dioxide (TiO_2_) with silver or functionalized graphene, got the particular attention.^128^ Upon exposure to light (UV, Solar), they generate ROS that break down microplastics into harmless byproducts like CO_2_ and H_2_O.^129,130^ Magnetic nanocomposites, especially those combining with iron oxide (Fe_3_O_4_), are also transforming environmental remediation by providing separable and reusable support systems.^131^ These materials effectively immobilize catalytic enzymes like PETase for targeted polymer degradation. They can be fascinatingly recovered after use. This offers a more sustainable and cost-effective alternative to enzymes.^132–135^

One of the polished steps in advanced wastewater treatment is the membrane modified with nanocomposites. This will help to remove microplastics through a dual mechanism: physical sieving combined with either chemical breakdown or surface adhesion.^136,137^ Even though there are challenges like membrane fouling, scalability, and high energy required for the removal of microplastics in complex wastewater.^138,139^ Addressing these issues through the integration of advanced photocatalytic, magnetic, and membrane functionalities within polymeric nanocomposites implies an auspicious multidisciplinary tactic for safeguarding aquatic ecosystems and human health.^140^

### 3.1 Carbon-based nanocomposite membranes

The efficiency of carbon-based nanomaterials in adsorption is determined by their tailored surface chemistry and porous structure.^141^ Graphene oxide, for instance, features a layered structure with oxygen functional groups that enable the hydrophilicity and further modification.^142,143^ Its affinity for microplastics arises from non-covalent interactions, predominantly π–π interaction between the GO lattice and aromatic plastic polymers, along with hydrophobic interactions with non-polar chains.^110,144,145^ Other than GO and CNT, modified biochar offers a sustainable and multifunctional platform.^146,147^ Enrichment of its surface area and active sites improves its capacity for microplastic removal from wastewater.^148^ Moreover, when this is applied in soils, it provides dual benefits: long-term carbon sequestration and soil improvement through better water retention, nutrient availability, and structure.^149,150^ More advanced applications involving carbon-based nanocomposite membranes are integrating CNTs and TiO_2_ into a polymer matrix *via* electrospinning or phase inversion.^151,152^ Electrospinning creates fibrous mats with high surface area for contaminant capture. However, phase inversion forms asymmetric membranes for controlled permeability.^153,154^ The synthesis process is very critical because the final product morphology directly impacts membrane performance and durability.^155^

The primary mechanism of CNT-TiO_2_ membranes combines physical retention and catalytic degradation.^156^ CNTs efficiently adsorb microplastics due to their electron-rich, hydrophobic surface. While TiO_2_ nanoparticles, upon light activation, generate ROS that break down the captured polymers.^121,157,158^ Complexity (precursor should be homogeneous) is the main limitation in this process. If the precursor is not homogeneous, then the synthesized nanocomposite material will agglomerate, and the surface area and efficiency will decrease.^159,160^ Membrane formation is also irreversible, and the need for high-purity precursors and energy-intensive processes raises questions about economic feasibility.^161,162^

### 3.2 Metal-oxide-based photocatalysts

In the advanced oxidation process, photocatalytic degradation significantly impacts the remediation of microplastics from wastewater.^163^ Using semiconductor materials like ZrO_2_, TiO_2_, CeO_2_, MgO, and ZnO as catalysts will enhance the efficiency.^164^ The fundamental mechanism of photocatalysis is that light irradiation of the photocatalyst degrades impurities.^158^ The main criterion for selecting the light source energy should be equal to or greater than the band gap of the catalyst. This light promotes the electron to the conduction band and generates positively charged holes in the valence band.^165^ This electron hole pair separation initiates the reaction with the water and oxygen, which leads to the formation of the highly reactive oxygen species.^166^ In recent years, researchers have been mainly focused on how to enhance the efficiency of these photocatalysts through modifications.^122,167^

**Fig. 5:**
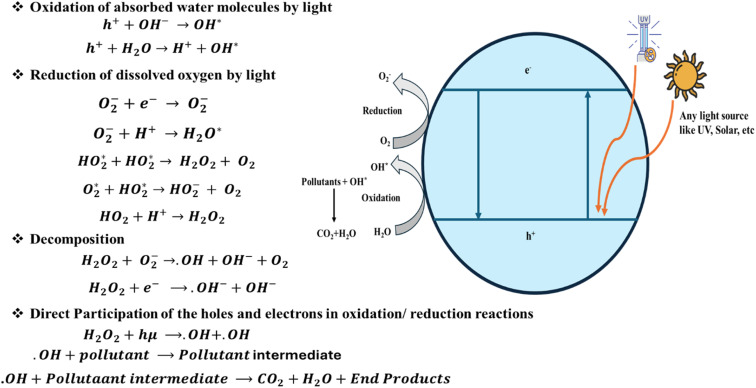
General schematic representation of the photocatalytic degradation process.

This is mainly aimed at improving the charge separation and extending light absorption into the visible spectrum.^159^ For example, the synthesis of TiO_2_-doped semiconductor nanocomposites has achieved almost complete mineralization of the polymer chains in wastewater.^156^ Under the optimized laboratory conditions, these advanced nanocomposites have shown a high efficiency of about 98.4% degradation.^130,168,169^Fig. 5 shows the general schematic representation and reaction mechanism of how the photocatalytic degradation takes place in the presence of different light sources, such as UV, solar, *etc.*

Despite the promising efficiency in controlled settings, significant challenges delay the transition of photocatalytic nanotechnology to large-scale environmental applications.^120^ A primary limitation is the reliance on specific light activation; many photocatalysts require ultraviolet light for optimal performance, necessitating energy-intensive illumination sources.^170^ While strategies to develop visible-light-active catalysts are underway, the subsequent hurdle of recovering and reusing nanoscale particles from treated aqueous contaminants remains a critical and economic barrier.^161^ This difficulty in catalyst separation poses a risk of secondary pollution, and it will naturally increase operational costs.^171^ The table (Table 1) given below shows some of the metal oxide nanocomposites used as the catalyst for the degradation process.

**Table 1:** Efficient metal oxide nanocomposites used as the catalyst for the degradation process

Nanocomposite	Synthesis methods	Mechanism	Microplastics targeted	Performance efficiency	Ref.
CeO_2_–ZnO heterojunction	Co-precipitation	Electrostatic adsorption + enzymatic degradation	PET	>85% adsorption; PETase enables	172
TiO_2_–GO	Sol–gel/hydrothermal of Ti precursors in GO	Photocatalysis with ROS; improved charge separation	PE, PS, PP	∼90% polymer mass loss under simulated sunlight	173
PANI–TiO_2_	Oxidative polymerization of aniline	Combined photocatalytic and electrochemical mechanism	PE, PS	80% PE and PS degradation under concurrent UV illumination and an applied bias	174
ZnO–Ag	Hydrothermal + Ag doping *via* photo-assisted deposition	Plasmon-enhanced visible-light photocatalysis + antibacterial	PE, PP	75% PE degradation in 6 h; microbial disinfection	175
TiO_2_/ZrO_2_ heterojunction	Sol–gel + hydrothermal method	Photocatalysis: UV light (365 nm), 6 h, catalyst loading 1 g L^−1^	PP	∼92% weight loss in 6 h; enhanced	176
TiO_2_–chitosan	Blending/casting TiO_2_ in crosslinked chitosan	Adsorption + photocatalytic degradation (UV)	PS, PET	>60% PS degradation after 12 h UV	177
Polyaniline–TiO_2_	*In situ* oxidative polymerization of aniline	Photoelectrochemical ROS generation	PE, PS	∼80% degradation *via* combined UV + bias	178
UiO-66/g-C_3_N_4_ heterostructure	*In situ* solvothermal growth of UiO-66 on g-C_3_N_4_	Piezocatalysis: ultrasonic irradiation	PS	∼85% degradation (based on TOC removal)	179
Co-doped ZrO_2_	Co-precipitation	Persulfate activation: peroxymonosulfate (PMS) activation	PS	94% degradation efficiency; catalyst stable for 5 cycles	180
N-doped ZrO_2_	Hydrothermal + post-annealing in NH_3_ atmosphere	Photocatalysis: visible light (*λ* > 420 nm), 12 h, catalyst 1 g L^−1^	PET	∼65% surface erosion	181
ZrO_2_/ZnO core–shell	Atomic layer deposition (ALD) of ZrO_2_ on ZnO nanorods	Photocatalysis: UV-A light, 8 h, catalyst 0.5 g L^−1^	PVC	88% chlorine release (measured by ion chromatography)	182
ZrO_2_–TiO_2_ @ rGO	One-pot hydrothermal synthesis	Filtration + photocatalytic degradation	PP	95% weight loss; rGO enhances electron transfer and MP adsorption	183

### 3.3 Magnetic iron oxide systems for microplastic remediation

In water treatment, the most persistent technical challenge is the cost-effective and efficient removal of the effluent.^184^ One of the best applications for the nanomaterial is water treatment. High surface area and reactivity make the nanomaterial more distinctive and effective for removing microplastics and contaminants from water. Because using normal and conventional filtration or sedimentation methods, it won't be removed.^163^ If it does not react properly, it will produce secondary pollutants and hinder their reusability.^185^ To overcome this issue, one innovative method is the use of magnetic iron-based nanoparticles, specifically magnetite (Fe_3_O_4_) and maghemite (γ-Fe_2_O_3_).^172^ These iron-based materials show a superparamagnetic nature, which means under an applied external magnetic field, they exhibit strong magnetization, enabling rapid and facile collection from aqueous suspension.^173^ Importantly, after the removal of the external magnetic field, they lose their magnetism and return to their original state. This is very critical for the creation of advanced nanocomposite adsorbents.^174^

A little more advanced version of these iron-based nanocomposites is the magnetic biochar. Which featuring a core–shell structure of magnetic iron oxide and a porous structure of biochar, which is produced from the biochemical reaction.^175^ This material is a highly efficient and sustainable solution for removing pollutants such as microplastics from water sources.^118^ This advanced material combines high surface area and functional groups of biomass-derived carbon with facile magnetic separation.^176^ This design produces a multifunctional material that can be arranged and saturated with contaminants, and after the reaction, can be efficiently recovered and regenerated.^135^ Nowadays, this magnetic biochar is dramatically improving its process economics and practicality. Because this has inherent benefits of biochar, such as extensive surface area, a complex porous network, surface functional groups, and low-cost precursor materials, while gaining the novel capability of magnetic separation.^131^ By integrating magnetic responsiveness with superior adsorption, this scalable, sustainable approach significantly enhances operational practicality compared to conventional materials.^132^

Considering this foundation, the researchers are now concentrating on developing these types of advanced magnetic nanocomposites to remove the microplastic pollution from the aquatic environment.^124^ These particles are designed with a special structure so that they can actively destroy the captured microplastic.^177^ These shells can be activated by chemical reactions like photocatalysis, and this will produce the reactive oxygen species (ROS), which break down the polymer chains into harmless components.^121^ The following table (Table 2) synthesizes findings from recent studies, outlining several composite materials and their documented performance in microplastic remediation, illustrating the progression from simple magnetic recovery to integrated capture-and-destroy technologies.

**Table 2:** Fascinating magnetic iron oxide-based nanocomposite catalyst and its efficiency for microplastic remediation

Nanocomposite	Synthesis highlights	Mechanism	Targeted microplastic	Efficiency	Ref.
Fe_3_O_4_-polydopamine	Magnetite core with polymer adhesive coating	Adhesion and magnetic separation	Polystyrene (PS) beads	∼92% removal	178
Co–Zn ferrite/peroxymonosulfate	Cobalt-zinc ferrite catalyst	Advanced oxidation + photocatalytic degradation	Polyethylene (PE) fragments	∼85% degradation (weight loss)	179
Magnetic biochar (Fe-BC)	Sludge-derived biochar with Fe_3_O_4_	Adsorption + photocatalytic degradation	Polyvinyl chloride (PVC)	>90% and removal from aqueous suspension	180
Fe_3_O_4_–SiO_2_–TiO_2_	Magnetic core, silica spacer, TiO_2_ shell	Adsorption + photocatalytic degradation	Polypropylene (PP)	∼88% degradation under UV light	181
Fe_3_O_4_–C/ZnO	Magnetic carbon core with ZnO	Adsorption + photocatalytic degradation	Polystyrene (PS)	∼95% enhanced breakdown	182
LaFeO_3_ perovskite	Lanthanum ferrite (magnetic perovskite)	Catalytic degradation *via* peroxymonosulfate	Polyethylene terephthalate (PET)	∼80% catalytic degradation	183
Fe_3_O_4_@MIL-101(Fe)	Magnetic core with metal–organic framework	Adsorption and fenton-like degradation	Nylon fragments	∼89% degradation	186
NiFe_2_O_4_/UV/PMS	Nickel ferrite catalyst	Synergistic UV/peroxymonosulfate process	Polyethylene (PE) powders	∼87% degradation	187

This technological path, from magnetic adsorbents to catalytic nanocomposites, addresses the critical recovery limitation while adding transformative functionality. It represents a significant step toward deployable, sustainable, and closed-loop nanomaterial-based systems for complex water pollution challenges. Future development focuses on enhancing the specificity, stability, and catalytic strength of the shells, and assessing the long-term performance and environmental fate of these engineered composites in real water sources.^44^

### 3.4 Biodegradable polymer-based nanocomposites

The propagation of microplastics (MPs) in aquatic and global ecosystems represents a persistent environmental challenge.^188^ So nowadays the whole world is demanding remediation methods which should be effective as well as ecologically liable.^189^ In the traditional methods, which often rely on non-biodegradable or chemical catalysts. But after use, it may produce secondary pollutants or require complex recovery processes.^163^ To overcome this limitation, materials science focused on the development of advanced materials composites, where active catalytic or adsorptive nanoparticles are combined within biodegradable polymer matrices.^105^ These composites are modified to execute the dual function of capturing and degrading pollutant particles. While ensuring that they themselves undergo environmental degradation.^107^

These biodegradable advanced materials typically utilize natural or synthetic bio-based polymers like cellulose, chitosan, polylactic acid (PLA), *etc*, as supporting frameworks.^190,191^ In this process, the choice of polymer is critical. Because it provides not only structural integrity but also intrinsic functional groups like amino groups in chitosan and carboxyl groups in alginate. This can enhance the adsorption of MPs *via* electrostatic interactions or hydrogen bonding.^117^ By immobilizing or embedding photocatalysts like TiO_2_, ZnO, Fe_3_O_4,_ or enzymes (PETase) within these matrices, a harmonious remediation mechanism is achieved.^192,193^ The polymer matrix first concentrates MPs from the aquatic medium, thereby increasing their local concentration at the active sites. Where the resultant degradation occurs through radical attack or enzymatic hydrolysis, photocatalytic oxidation.^121^

One of the vital advantages of this process is its inherent sustainability and reduced environmental footprint.^184^ In this, a biodegradable polymer component is intended to decompose into non-toxic byproducts under ecological conditions, unlike the traditional plastic-based filters or inorganic substrates.^190^ Moreover, this advanced material was synthesised for the recovery of microplastics *via* magnetic separation. Sufficiently encapsulated to prevent its own nanotoxicity, leaching, *etc.*^177^ Mainly, this approach fits with the economic objectives like reusable precursors, reusable materials, and is also designed for the end of biodegradability.^121^ Even if this field is moderately new, it represents a promising pathway toward more sustainable environmental management technologies.^189^ The table (Table 3) summarizes notable examples of efficient biodegradable polymer-based hybrid systems developed for microplastic degradation.

**Table 3:** Efficient biodegradable polymer-based hybrids for microplastic degradation

Nanocomposite	Synthesis methods	Mechanism	Targeted microplastic	Efficiency	Ref.
Chitosan/TiO_2_ composite film	Solvent casting & cross-linking	Adsorption + photocatalysis (UV)	PS	>60% PS degradation in 12 h; chitosan enhances MP adsorption	194
PLA/Fe_3_O_4_ nanofibrous mat	Electrospinning	Adsorption + fenton-like catalysis (with H_2_O_2_)	PE	∼75% PE degradation in 6 h; magnetic recovery of catalyst	192
Cellulose nanofiber/ZnO aerogel	Freeze-drying & *in situ* growth	Adsorption + photocatalysis (visible light)	PP	85% removal efficiency; superhydrophilic for oil/water separation	195
Chitosan/enzyme (PETase) beads	Ionic gelation	Enzymatic hydrolysis	PET	Significant surface erosion & weight loss; reusable for multiple cycles	196
Alginate/CeO_2_ microbeads	Ionotropic gelation	Photocatalysis + peroxymonosulfate activation	PS	∼90% degradation *via* radical oxidation; bead stability maintained	197
Starch/Ag–TiO_2_ porous foam	Thermal processing & doping	Adsorption + plasmonic photocatalysis	PE, PS	∼80% degradation under solar simulator; antibacterial properties	198
PHA/TiO_2_ composite nanoparticles	Emulsion-solvent evaporation	Photocatalysis (UV)	PVC	Enhanced dechlorination rate; PHA shell controls release	199
Gelatin/ZnFe_2_O_4_ hydrogel	Cross-linking & co-precipitation	Adsorption + photo-fenton	PE	70% degradation under visible light; excellent reusability	200
Lignin-derived carbon/Fe composite	Pyrolysis & impregnation	Adsorption + reductive degradation	PS	High adsorption capacity with subsequent reductive breakdown	44
Cyclodextrin-based polymer/GO foam	Cross-linking & assembly	Adsorption + peroxydisulfate activation	Various MPs	>95% removal *via* adsorption-catalysis synergy; exceptional selectivity	201

### 3.5 Computational insights into nanocomposite performance

Most of the studies in the domain of microplastic remediation using nanocomposites are based on experimental methodologies. But in recent years, computational methods have been increasingly used to understand the fundamental interactions that influence nanocomposite performance in microplastic cleanup. Notably, density functional theory (DFT) helps estimate how strongly polymer chains interact with catalyst surfaces, providing detailed molecular insights into adsorption processes. In the case of binding affinity, these calculations expose that even delicate variations in surface crystallography, elemental composition, and functional groups can cause significant differences. Subsequently, such perceptions help to explain why certain nanocomposites exhibit superior performance in microplastic degradation. It also explains the empirical efficiency comparisons towards the mechanistic understanding of structure–property relationships.^202–205^

Meanwhile, molecular dynamics (MD) simulations offer a deeper understanding of interfacial phenomena, especially involving aromatic polymers and carbon-based nanomaterials. It cannot be easily captured through static quantum mechanical calculations; it needs advanced knowledge of dynamic interfacial processes. MD simulations allow observation of the time-dependent behavior of polymer chains in proximity to nanomaterial surfaces, thereby offering a more realistic representation of environmental conditions. This study is mainly important in the case of complex polymer material which containing aromatic groups. This study indicates that non-covalent interactions like π–π stacking significantly stabilize polymer adsorption and correlate well with experimental data. MD results also reveal that polymer chain flexibility is crucial, with more flexible chains maintaining longer contact with surfaces than more rigid structures.^202–205^ The rigid polymer structure exhibits limited conformational adaptability. This will lead to a weaker and more transient interaction. This discrepancy is predominantly relevant when considering the heterogeneous nature of environmental microplastics, which vary widely in composition, crystallinity, and degree of weathering. When considering this type of material, the computational approaches offer a powerful framework for predicting performance trends across different material systems.^202–205^

When considering both DFT and MD simulations, they not only bridge the gap between experimental observations and theoretical understanding but also provide a predictive platform for the balanced design of next-generation nanocomposites. These methods help to enable researchers for the systematic study the material combinations, optimize surface functionalities, and tailor interfacial properties to enhance microplastic remediation efficiency. As computational methods and methodological cleverness continue to advance, their role in guiding experimental research and accelerating innovation in the degradation field using nanocomposites is expected to become increasingly necessary.^202–205^

## 4. Performance, sustainability, and implementation challenges

Recent laboratory-scale investigations have established that a wide range of nanocomposite-based materials can achieve high removal efficiencies for microplastics and related contaminants. It typically ranges from approximately 73% to 99% in both simulated samples and real water samples.^123^ These promising results show that the strong affinity and tunable surface properties of advanced nanomaterials can be achieved under controlled experimental conditions.^138^ After a closer evaluation of recent studies on nanocomposite-based materials for the microplastic degradation, several inconsistencies are exposed. The first one is the influence of the incorporation of TiO_2_ in polymer-based systems. Multiple reports indicate that advanced nanoparticle loadings enhance the photocatalytic degradation efficiency. While some other reports indicate moderated performance at higher concentrations, which has been attributed to reduced light penetration, altered polymer crystallinity, and particle agglomeration. But these discrepancies are rarely resolved because key parameters, like interfacial compatibility, dispersion quality, and crystallinity indices, are not consistently quantified.^205–208^

**Fig. 6:**
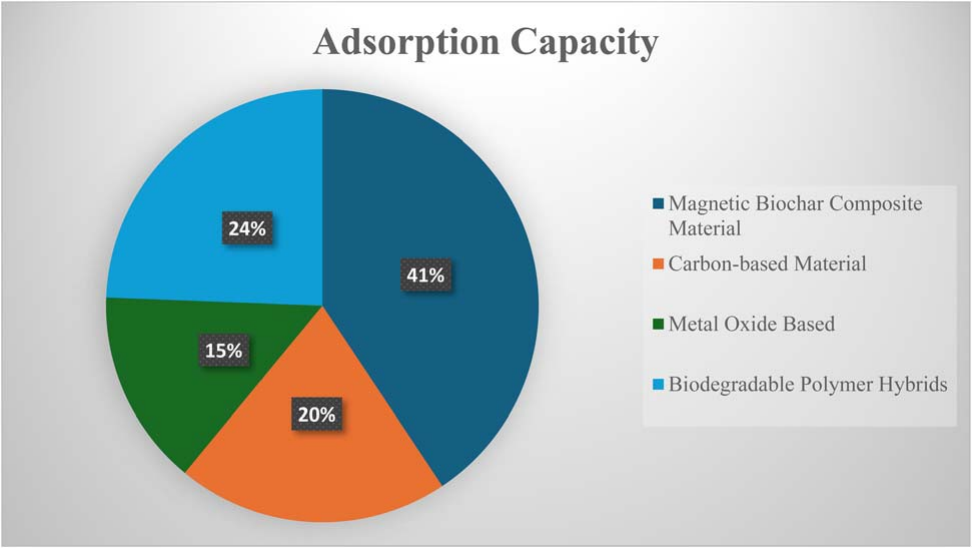
Adsorption percentage of microplastics from wastewater using different advanced materials.

Another area of disagreement involves the relative performance of nanomaterials like ZnO and TiO_2_ under visible-light irradiation. In the ZnO-based material, more often reported to exhibit higher activity due to its favourable charge transport properties. However, several studies demonstrate that doped TiO_2_ can outperform ZnO by extending light absorption into the visible region and suppressing electron–hole recombination.^209–211^ This consideration study suggests that photocatalytic efficiency is mainly ruled by the surface modifications and defect engineering, and less by intrinsic band gap values. Another one is that the pH range remains poorly resolved. In the optimal conditions, pH varies from acidic to neutral to alkaline environments. This mainly depends on catalyst composition, surface charge characteristics, and the dominant ROS involved in the degradation pathway. Such changeability indicates that pH effects are mainly system-specific rather than universal.^212^ Collectively, these inconsistencies underscore the absence of harmonized testing protocols and highlight the importance of comprehensive material characterization to enable meaningful comparison across studies. In Fig. 6 shows the adsorption percentage of microplastics from wastewater.

When evaluated under real-world conditions, several practical limitations emerge.^213^ Adsorbent regeneration and reusability tend to decline after repeated use, leading to diminished efficiency and higher material consumption.^214^ Additionally, the long-term stability of these nanocomposites in natural water matrices where pH, salinity, organic matter, and pollutant mixtures vary remains insufficiently characterized.^118^ Another major issue concerns the environmental safety of the nanomaterials themselves.^185^ The potential release of modified nanoparticles during production, application, or disposal raises concerns about their persistence, bioavailability, and ecological toxicity.^215^ These nanoparticles may affect aquatic organisms across different trophic levels. It causes oxidative stress, accumulation, or chronic effects.^216^ Hence, before the large-scale deployment, there should be a very rigorous investigation needed about the secondary pollution risks, performance beyond controlled conditions, and long-term ecological impacts.^217^

Life-cycle assessment has become an important tool for evaluating the sustainability of nanocomposite-based recommendation methods.^218^ According to the existing studies of life cycle assessment, the biochar-based materials perform more favourable to the microplastic removal. Which is typically showing net carbon emissions between −2 and +5 kg CO_2_ eq per kg of plastic removed.^146^ In addition, GO-based nanocomposites convey a heavier environmental trail, with estimated emissions ranging from 25 to 60 kg CO_2_ eq per kg of microplastics removed. This is mainly due to the energy-intensive synthesis and chemical processing.^219^ The lack of consistent testing protocols makes them difficult for further meaningful evaluation. It slows down the transition from laboratory research to accountable field applications.^121^

## 5. Concluding remarks and future perspectives

From a future research perspective, bridging the gap between laboratory-scale methods and real-world applications requires a strategic reorientation.^127^ One of the most promising designs is the multifunctional nanomaterials that integrate adsorption, catalytic degradation, and magnetic-assisted recovery within a single material dais.^126^ The main advantage of such materials is to minimize process complexity, operational costs, and boost overall treatment efficiency. It concurrently captures and transforms the microplastics into nontoxic elements like CO_2_ and H_2_O.^124^ Under the dynamic conditions, the materials should increasingly emphasize their durability, reproducibility, and performance stability. They should also ensure that laboratory-based advantages are sustained during industrial-based long-term operation in various water sources.^170^

Another equally important aspect is the development of a standardized protocol and evaluation framework.^183^ Currently, it is very challenging to directly compare efficiencies across reported studies because of the wide variety of experimental methods, microplastic characteristics, and wastewater treatment applications.^130^ Standardized materials that consider real-time environmental factors, particle size distribution, and the impact of secondary pollutants would allow for more consistent evaluations and processes.^122^ Within this standardized framework, integrating AI and machine learning tools offers significant opportunities to accelerate material discovery and process optimization.^220,221^ For this, the best example is that artificial neural networks have been successfully applied to predict the adsorption removal efficiency of polystyrene nanoplastic using a CoFe_2_O_4_ spinel-activated carbon composite, achieving exceptional predictive accuracy with test *R*^2^ values exceeding 0.967.^222^ Like that, there is a lot more work done using this AI and ML in the field of microplastic removal. Additionally, machine learning frameworks have been demonstrated for predicting adsorption efficiency on carbon-activated nanomaterials, addressing the inherent complexity of multicomponent adsorption systems.^223–229^ Data-driven approaches can identify structure performance relationships, predict long-term behaviour, and guide the rational design of next-generation systems with less trial-and-error trialing.^224–230^ Future research should therefore prioritize the development of curated datasets linking nanomaterial physicochemical characteristics to microplastic remediation performance, followed by the deployment of supervised and unsupervised learning models to guide synthesis protocols and predict removal efficiencies under diverse environmental conditions.

From a broader sustainability perspective, future strategies must align microplastic remediation techniques with global economic principles. Rather than viewing the captured microplastics just as waste, emerging approaches could focus on their conversion into value-added chemicals, fuels, or composite materials for the different applications, like construction, infrastructure, *etc.* Such resource recovery pathways have the potential to offset treatment costs and reduce the environmental impact of remediation processes.^231^ Eventually, the successful transformation of microplastic removal technologies will not only depend on the technical efficiency but also on their economic feasibility, environmental safety, and integration within sustainable resource management contexts.^184^

## Author contributions

Veena C. G.: writing (original draft), investigation, visualisation, conceptualization and methodology, Chitra D.: conceptualization, review, editing and supervision.

## Conflicts of interest

There are no conflicts of interest declared.

## Data Availability

In this review, no primary research results have been included, and no new data were generated or analysed. All the data used in this study are already published. And all the references are correctly cited within the manuscript.
